# Biomechanical evaluation of a modified intramedullary nail for the treatment of unstable femoral trochanteric fractures

**DOI:** 10.1016/j.heliyon.2024.e29671

**Published:** 2024-04-14

**Authors:** ChaoFeng Wang, MingMing Hou, CongMing Zhang, Teng Ma, Zhong Li, Hua Lin, Kun Zhang, Qiang Huang

**Affiliations:** Department of Orthopedics, Hong Hui Hospital, Xi'an Jiaotong University, Xi'an, Shaanxi, 710054, China

**Keywords:** Finite element model, Modified, Intramedullary nail, Unstable, Trochanteric fracture

## Abstract

**Background:**

The Proximal Femoral Nail Antirotation (PFNA) device is the most commonly used implant to fix unstable femoral trochanteric fractures (UFTFs), but it has a relatively high incidence of complications. Due to this factor, the modified intramedullary nail (MIN) was created to treat patients with UFTFs. The aim was to exhibit the MIN and make a comparison with PFNA and InterTAN using biomechanical methods.

**Methods:**

An adult UFTF model was developed using Mimics software. The PFNA, InterTAN nail, and MIN models were drawn referring to the corresponding parameters and installed in the fracture models. Vertical, anteroposterior (AP) bending, and torsion loads of the femoral head were set in advance and loaded onto the fracture models. The value of maximal displacement and von Mises stress was evaluated via finite element analysis (FEA).

**Results:**

The MIN model had smaller values for maximal displacement than that of the PFNA model, and the increase in displacement was less pronounced for the MIN compared to PFNA under increasing vertical loads. For the indicator of von Mises stress, the MIN model showed lower stress compared with the PFNA model in vertical loads ranging from 300 N to 2100 N. Except for the maximal stress at implants under AP bending loads, the MIN demonstrated the most superior biomechanical properties under AP bending and torsional loads.

**Conclusion:**

The MIN offered obvious advantages in terms of mechanical stability and stress distribution among the three studied implants, providing a promising implant option for patients with UFTFs.

## 1 Introduction

Femoral trochanteric fractures mainly occur in the elderly, especially those with osteoporosis, which poses a significant threat to their health. Femoral trochanteric fractures account for 3.4 % of all fractures and approximately 1.6 million cases occur annually worldwide, projecting to rise to 6.3 million by the 2050s [[1], [2], [3]]. Patients with such fractures face a high rate of postoperative complications and mortality within one year, with the latter ranging from 11 % to 29 % [4,5]. Moreover, most of these fractures turn out to be unstable femoral trochanteric fractures (UFTFs) [6]. Comminuted trochanteric fractures make up roughly 80 % of all UFTFs, particularly those classified as AO/OTA 31-A2.3 [7]. The standard treatment emphasizes surgical intervention within 48 h to promote functional recovery and reduce complications associated with prolonged bed rest [8].

Notably, intramedullary fixation is recommended for unstable trochanteric fractures according to the 2018 Osteosynthesefragen/Orthopaedic Trauma Association (AO/OTA) guideline. Commonly used intramedullary implants for treating these fractures include the PFNA device, the Gamma3 nail, and the TriGEN InterTAN nail [9]. These implants are fixed axially and allow for early weight-bearing. PFNA has been widely used and its helical blade can increase the stability of the bone-implant interface by compacting cancellous bones [10]. Unlike PFNA, the clinical application and literature reports of Gamma3 are limited. However, Bonnaire et al. demonstrated that Gamma3 and the PFNA device achieved similar clinical effects for treating UFTFs [11]. Both Gamma3 and PFNA are considered to be single-screw intramedullary implants, but their antirotation capacity is insufficient. This disadvantage can be further amplified in unstable fractures, increasing the risk of implant failure. In contrast, InterTAN provides inter-fragment compression and locking through an integrated double-screw configuration [12]. Biomechanical testing has shown that the double-screw InterTAN device has better antirotation capacity, higher biomechanical strength, and can bear larger loads compared to single-screw devices [13]. Furthermore, patients using the InterTAN nail experience faster union, better clinical outcomes, and lower postoperative complications in clinical practice [14,15]. Nevertheless, the parrel neck screws are positioned very closely together for the InterTAN nail, almost resembling one “thicker” screw. As a result, its antirotation effects are still limited and it cannot provide sufficient support for medial cortical defects in unstable fractures.

**Fig. 1:**
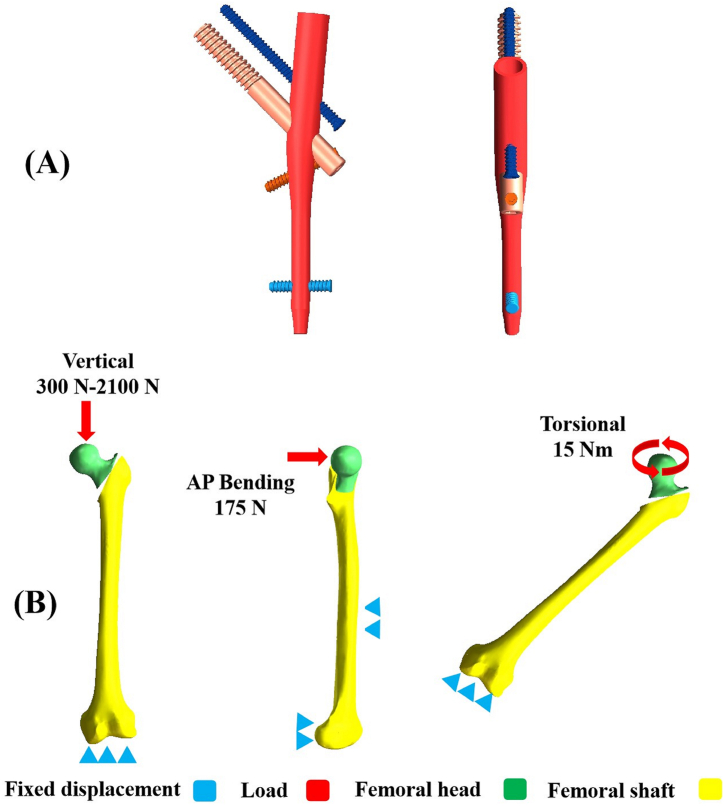
Schematic diagrams of the MIN and three boundary conditions. (A) Schematic diagrams of the MIN. (B) Boundary conditions for vertical, AP bending, and torsional loads. MIN stands for the modified intramedullary nail. AP stands for anteroposterior.

Due to the aforementioned factors, the modified intramedullary nail (MIN) was created to enhance the fixation effects of UFTFs. The innovation for the MIN lies in the inclusion of three screws at the proximal part. As shown in Fig. 1A, there are two neck screws in parallel arrangement. The lower cephalomedullary screw is responsible for compressing the fracture fragment, while the upper cephalomedullary screw primarily serves the antirotation function. Additionally, the subtrochanteric screw provides support for the medial cortex. Importantly, the interlocking mechanism of the neck screw and the subtrochanteric screw prevents the occurrence of the Z-effect that can arise with two parallel cephalomedullary screws. We anticipated that the MIN would offer superior biomechanical stability compared to traditional implants for treating UFTFs.

Since the 1950s, finite element analysis (FEA) has been initially used in the industrial field [16]. Subsequently, this technique became widely used in the orthopedic field for mathematically simulating von Mises stress and displacement based on material characteristics and loading boundary conditions. As a result, it has evolved into a promising method for identifying the properties of new implants or configurations [17,18]. In this study, the MIN was developed for fixing UFTFs via finite element settings. The mechanical properties of several implants, namely PFNA, InterTAN, and MIN, was compared under increasing vertical loads from 300 N to 2100 N, anteroposterior (AP) bending, and torsional loads. The values of displacement and von Mises stress were carefully evaluated.

## 2 Materials and methods

### 2.1 Finite element settings of implants and femur

Three-dimensional finite element model of femur was initially built using the CT data of a male volunteer who participated in this study voluntarily. CT data of the left leg were acquired. The 3D femoral model was created via aforementioned CT data. Hounsfield Unit (HU) values were utilized to make a distinction between cortex and cancellous bones, with a boundary set at 700 [17]. Following that, the AO/OTA 31-A2.3 UFTF model was developed by simulating two fracture lines based on previous literature [18,19]. This model represented a common type of comminuted and unstable trochanteric fractures with a posteromedial defect. The trochanteric crest, lesser trochanter, and part of the posterior greater trochanter were removed along the two osteotomy lines to simulate the unstable trochanteric fracture. The resulting 3D UFTF model was saved. Computer-aided design (CAD) software was used to visualize all 3D models of the implants. The key characteristics of PFNA and InterTAN implants were accurately simulated using manufacturer-provided data. The 3D UFTF model and the components of each implant were managed via a pre-processing software of FEA, and a Boolean operation was performed to assemble them together using LS-PrePost software (LSTC, Troy, MI, USA). Three fixation models for UFTFs were created, including the PFNA, InterTAN nail, and MIN models. Notably, schematic representations of the MIN are shown in Fig. 1A. The design parameters of MIN were shown as follows: 170 mm for the nail length, 17 mm and 10 mm for the proximal and distal diameters of the nail, 5 mm and 10 mm for the upper and lower cephalomedullary screw diameters, and 5 mm for the subtrochanteric screw diameter. Two parallel cephalomedullary screws were arranged uniformly in the femoral head and occupied the upper and lower one-third positions, respectively. The angle was designed as 130° between the cephalomedullary screws and the nail. Additionally, the included angle of the subtrochanteric screw and the nail was set at 70°. The subtrochanteric screw passed through the neck screw tail and the nail, and was fixed below the lesser trochanter. The tail of the subtrochanteric screw was locked with the lower cephalomedullary screw.

### 2.2 Finite element and boundary settings

Isotropic, homogeneous, and linear elastic material property was endowed to these bones as well as implants. The mesh type was defined as tetrahedral elements. The convergence tests were taken to determine the model reliability [18]. The field indexes of strain energy and displacement were set within an extent of five percent in terms of maximum Degree of Freedom. Moreover, maximum stress points were not allowed in the settings. Element and node values of the PFNA, InterTAN, and MIN models are exhibited in Table 1. All implants were assumed to be Titanium alloy for its superior biocompatibility, corrosion resistance, and ideal biomechanical property. The modulus of elasticity and Poisson's ratio for cortical and cancellous bones, as well as Titanium alloy, are described in Table 2 [18,20]. All contact situations, such as the contact between implants and femur, were considered to be frictional contacts. The frictional coefficient was set to 0.4 referring to similar literature [17]. The boundary conditions for three kinds of loads are shown in Fig. 1B. In vertical load case, the femur condyle had to be rigidly fastened to prevent any motion of the model. The increasing vertical loads loaded axially to represent vertical compression. For AP bending, both the shaft and condyle of the femur were completely fastened. The bending loads loaded laterally to the femoral head [18]. In the case of torsion, the torque force loaded to the femoral head along the femoral neck axis [18].

**Table 1:** Number of nodes and elements for three implant models.

Model	Nodes	Elements
PFNA	433466	280858
InterTAN	462455	293399
MIN	524693	334001

PFNA: proximal femoral nail antirotation. MIN: the modified intramedullary nail.

**Table 2:** Parameters of model materials.

Material	Elasticity modulus	Poisson's ratio
Cortical bone	16,800	0.3
Cancellous bone	840	0.2
Titanium alloy	110,000	0.3

### 2.3 Parameters for assessment and analysis

The analysis focused on the maximal displacement and stress distribution of implants when subjected to different loads. The trends of displacement and stress were also recorded as vertical loads increased. PFNA, which has been widely used as an intramedullary implant for treating UFTFs and has shown positive outcomes, was considered as the control group in this study. Variation rates (VR) were achieved as follows: VR=(V_1_ - Vn)/V_1_ × 100 %. Here, Vn means values of InterTAN or MIN, while V_1_ means values of PFNA.

## 3 Results

### 3.1 Maximal displacement under increasing vertical loads

**Fig. 2:**
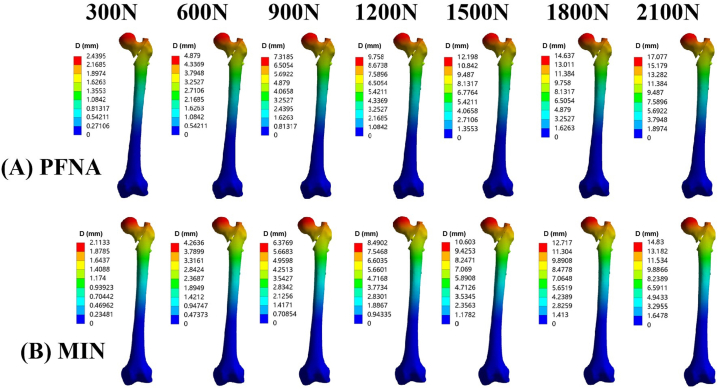
Cloud images of maximal displacement for PFNA and the MIN models under increasing vertical loads. (A) Maximal displacement for the PFNA model. (B) Maximal displacement for the MIN model. The increasing loads included 300 N, 600 N, 900 N, 1200 N, 1500 N, 1800 N, and 2100 N. PFNA stands for proximal femoral nail antirotation. MIN stands for the modified intramedullary nail.

**Fig. 3:**
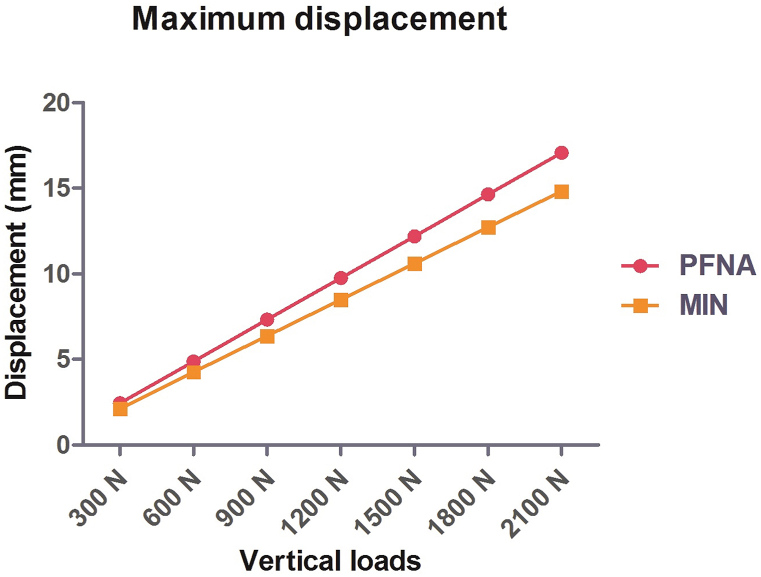
Line chart of maximal displacement for PFNA and the MIN models under increasing vertical loads from 300 N to 2100 N. PFNA stands for proximal femoral nail antirotation. MIN stands for the modified intramedullary nail.

Under the increasing vertical loads from 300 N to 2100 N, the maximal displacements for the PFNA group were recorded as 2.44 mm, 4.88 mm, 7.32 mm, 9.76 mm, 12.20 mm, 14.64 mm, and 17.08 mm, respectively (Fig. 2A); the maximal displacements for the MIN group were recorded as 2.11 mm, 4.26 mm, 6.38 mm, 8.49 mm, 10.60 mm, 12.72 mm, and 14.83 mm, respectively (Fig. 2B). Notably, as the vertical loads increased, the maximal displacement gradually increased for both the PFNA and MIN groups. However, the incremental trend of maximal displacement for MIN models was lower compared to those of PFNA models when subjected to increasing vertical loads. The cloud images illustrating the maximal displacement can be seen in Fig. 2, while Fig. 3 displays a line chart of displacement values.

### 3.2 Maximal stress of implants under increasing vertical loads

**Fig. 4:**
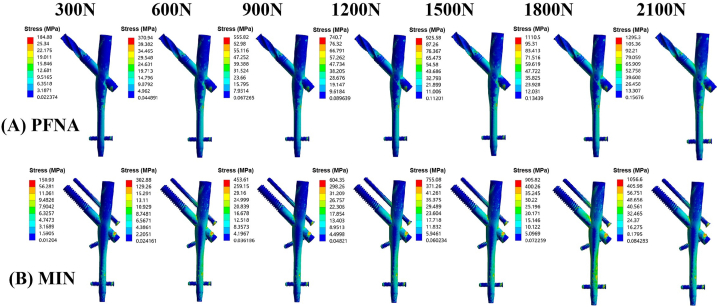
Cloud images of maximal stress at implants for PFNA and the MIN models under increasing vertical loads. (A) Maximal stress at implants for the PFNA model. (B) Maximal stress at implants for the MIN model. The increasing loads included 300 N, 600 N, 900 N, 1200 N, 1500 N, 1800 N, and 2100 N. PFNA stands for proximal femoral nail antirotation. MIN stands for the modified intramedullary nail.

**Fig. 5:**
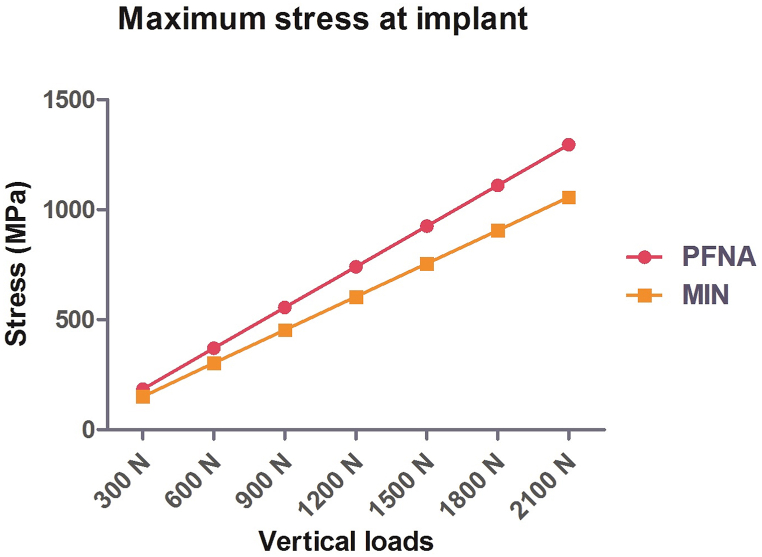
Line chart of maximal stress at implants for PFNA and the MIN models under increasing vertical loads from 300 N to 2100 N. PFNA stands for proximal femoral nail antirotation. MIN stands for the modified intramedullary nail.

Under the increasing vertical loads, stress was concentrated at the intersection between the neck screw and the nail in the PFNA group. For MIN, the area between the lower cephalomedullary screw and the nail was stress concentrated. As the vertical loads increased from 300 N to 2100 N, maximal stress values on implants for the PFNA group were recorded as 184.88 MPa, 370.94 MPa, 555.82 MPa, 740.70 MPa, 925.58 MPa, 1110.5 MPa, and 1295.3 MPa, respectively (Fig. 4A). Conversely, for the MIN group, the maximal stress values were 150.93 MPa, 302.88 MPa, 453.61 MPa, 604.35 MPa, 755.08 MPa, 905.82 MPa, and 1056.6 MPa, respectively (Fig. 4B). Notably, as the vertical loads increased, the maximal stress gradually rose in both groups. However, the incremental trend of maximal stress values for MIN models was lower compared to those of PFNA models under increasing vertical loads. Fig. 4 displays the cloud images illustrating the maximal stress, while Fig. 5 shows a line chart representing this parameter.

### 3.3 Maximal displacement under AP bending and torsional loads

**Fig. 6:**
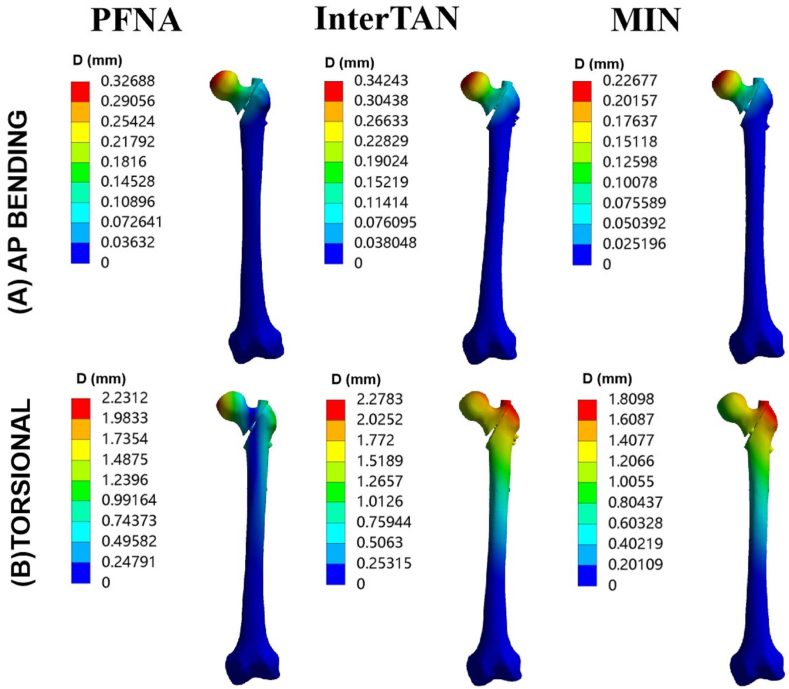
Maximal displacement for three implant models under AP bending and torsional loads. (A) Maximal displacement for three implant models under AP bending loads. (B) Maximal displacement for three implant models under torsional loads. Three models included PFNA, InterTAN and the MIN. AP stands for anteroposterior. PFNA stands for proximal femoral nail antirotation. MIN stands for the modified intramedullary nail.

The cloud images showing the maximal displacement in AP bending and torsion loads can be seen in Fig. 6. When subjected to AP bending with a force of 175 N, the maximal displacement values of three fixation models were recorded as 0.33 mm, 0.34 mm, and 0.23 mm, respectively (Fig. 6A). Additionally, in torsional load case, the maximal displacement values were recorded as 2.23 mm, 2.28 mm, and 1.81 mm for PFNA, InterTAN, and the MIN, respectively (Fig. 6B). Comparing the results to the PFNA model, the MIN model showed a reduction rate of maximal displacement by 30.6 % in AP bending load case and 18.9 % in torsional load case.

### 3.4 Stress at implants under AP bending and torsional loads

**Fig. 7:**
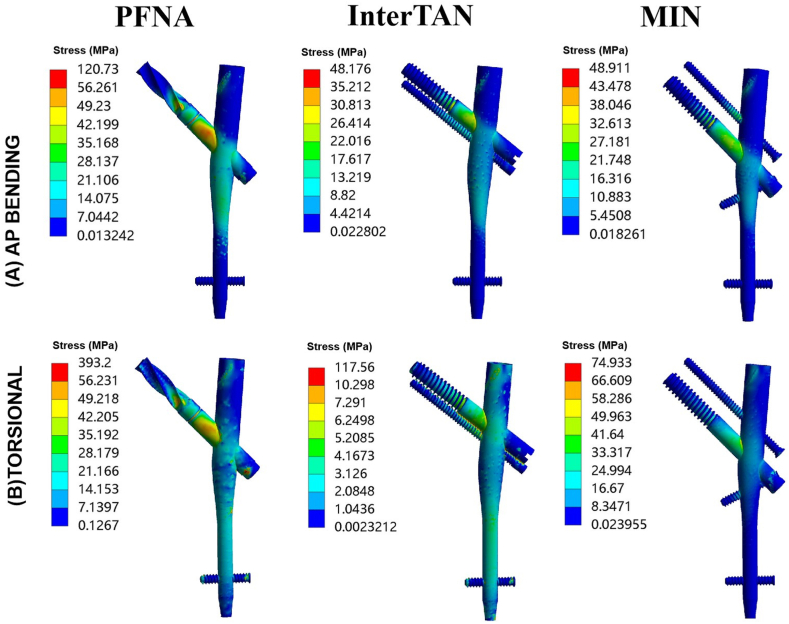
Von Mises stress at implants for three implant models under AP bending and torsional loads. (A) Maximal stress at implants for three implant models under AP bending loads. (B) Maximal stress at implants for three implant models under torsional loads. Three models included PFNA, InterTAN and the MIN. AP stands for anteroposterior. PFNA stands for proximal femoral nail antirotation. MIN stands for the modified intramedullary nail.

The cloud images depicting the maximal stress values at implants in AP bending and torsion load cases are presented in Fig. 7. When subjected to AP bending loads, according to the maximal stress values the fixation models can be ranked as follows: PFNA, the MIN, and InterTAN (Fig. 7A). In torsional load case, the ranking of the three fixation models was PFNA, InterTAN, and the MIN (Fig. 7B). The value of maximal stress for the MIN was lower compared to that of the PFNA in both AP bending and torsional load cases. The VR reduction of maximal stress for the MIN relative to the PFNA was 59.5 % in AP bending load case and 80.9 % in torsion load case.

## 4 Discussion

This study was aimed to introduce a MIN for the treatment of UFTFs under increasing vertical, AP bending, and torsional loads. Our data demonstrated that the MIN owned superior mechanical stability compared to the PFNA and InterTAN nail.

Several scholars have made efforts to design new types of implants for the improved treatment of UFTFs. One such innovation is the medial sustain nail, which is a newly designed cephalomedullary nail that restricts sliding to decrease the implant failure rate of UFTFs [19]. Nie's study demonstrated that the medial sustain nail had more superior mechanical stability than the PFNA device, and it may effectively prevent femoral medialization and cut-out [19]. This new implant consists of two cephalomedullary screws. The upper screw fixes and compresses the fragment, while the lower screw provides medial support. However, due to its short length, the lower screw's antirotation capacity is inadequate. Another implant called Proximal Femoral Bionic Nail (PFBN) was developed to fix trochanteric fractures based on a triangular stabilization configuration and lever-balance reconstruction theory [21,22]. The PFBN device is composed of a main nail and two cephalomedullary screws. The two neck screws are called the tension screw and the pressure screw, respectively. These three components form a relatively stable structure. A recent research demonstrated that the PFBN device had more superior biomechanical properties than the PFNA and InterTAN implants [23]. Chen et al. also found that, in patients with revised trochanteric fractures, the PFBN provided greater mechanical support than the PFNA and DHS devices [24]. However, the medial cortex support offered by the PFBN is limited, and its ability to provide effective fixation for unstable trochanteric fractures needs further verification. Restoring medial cortex support is a crucial step in the treatment of UFTFs but can be challenging during surgery. The majority of unstable trochanteric fractures involve defects in the medial cortex, which is a significant contributing factor to implant failure. According to Chen's study, the incidence of reduction loss due to comminuted medial wall fractures after surgery reached up to 20 % [25]. Therefore, improving medial support through modified implants may be a promising approach to address UFTFs. Based on these considerations, our team has designed a MIN to better stabilize UFTFs.

FEA has been introduced in orthopedic research for decades. It can accurately depict the minor changes in mechanics caused by the insertion of an implant through mathematical simulation, thus assisting in explaining how the bones influence these changes under different loads and boundary conditions [26]. Additionally, it allows for modifications to implant properties and facilitates evaluation of the most exceptional devices. Our team used FEA simulation to create UFTF models and conducted tests on the corresponding mechanical properties of several implants. The results demonstrated that, the MIN showed more superior mechanical stability among the three studied implants when subjected to increasing vertical, AP bending, and torsional loads. These findings might be attributed to the modifications made to the MIN as compared to traditional intramedullary implants. Traditional implants, comprising Reconstruction Nail (RA), the Proximal Femoral Nail (PFN), etc. also employ two parallel cephalomedullary screws in their design. The MIN shares certain similarities with the reconstruction nail and the PFN in this aspect. However, under increasing vertical loads, the upper cephalomedullary screw of the reconstruction nail and the PFN may cut out, while the lower cephalomedullary screw may retract, leading to a Z-effect that often causes implant failure [27,28]. The primary difference and innovation of the MIN lies in the introduction of a subtrochanteric screw. This screw is securely locked with the lower cephalomedullary screw in a different direction. Such a design effectively reduces displacement and minimizes the incidence for the Z-effect or reverse Z-effect, thereby providing superior biomechanical stability.

For the PFNA and InterTAN nails, the intersection of the nail and the neck screw is a stress concentration area. Yet, as maximal stress results showed, the integrated MIN was stress dispersed to some extent. This might result from the reasonable design of the MIN. From a geometric perspective, single (PFNA) or tight arrangement of InterTAN will lead to stress concentration. Dual-screw cephalomedullary nail of the MIN has a uniform geometric arrangement. The dispersed arrangement of the dual-screw cephalomedullary nail (the MIN) will lead to stress dispersion. The smaller the stress borne by the internal implant, the less likely it is to break. Based on our data, compared to PFNA and InterTAN, the MIN had the lowest von Mises stress under increasing vertical, AP bending, and torsional loads. This also means that under the same loads, the risk of implant failure is minimized when using the MIN to treat UFTFs.

Previous studies have demonstrated the superior antirotation capabilities of InterTAN compared to PFNA for stabilizing UFTFs [[13], [14], [15]]. Our own research reaffirmed this finding. Furthermore, our results indicated that the MIN provided the best antirotation, followed by InterTAN, while PFNA had the weakest antirotation. Although both MIN and InterTAN possess a dual-screw structure, the arrangement of the screws differs. In InterTAN, the two cephalomedullary screws are tightly positioned, resembling a thicker single screw from a geometric perspective. Conversely, the two cephalomedullary screws of the MIN are evenly distributed and positioned within the femoral head. Additionally, the subtrochanteric screw is interlocked with the lower cephalomedullary screw. This unique design may explain why the MIN provides the best antirotation among these three implants. It is worth noting that UFTFs often involve a comminuted or even deficient medial wall [7]. Under the same loads, smaller displacement indicates better medial support provided by the implant. Compared to the PFNA and InterTAN, the MIN had the least displacement under three load cases. The inclusion of the subtrochanteric screw in the MIN may contribute to these results. The subtrochanteric screw potentially aids in reconstructing the medial wall and enhancing medial support to some extent. This could be considered an additional advantage of the subtrochanteric screw in the MIN.

This research still had several limitations. The finite element model simplified the real situation, and it did not consider the influence of soft tissues such as tendons, muscles, and fascia on implants. However, our study specifically focused on assessing the biomechanical stability of different implants. By excluding soft tissues, we maintained a clear and precise objective. Additionally, the femur materials were set as homogeneous as well as isotropic. This simplification of material properties was done to strictly control the variables and minimize interference in our conclusions. It was also an advantage of the finite element method compared to cadaveric bone experiments and clinical studies. Nevertheless, further research will be conducted to validate the effectiveness of this new implant using cadaver bones and patients.

## Conclusions

The MIN demonstrated clear benefits with regard to biomechanical stability and stress distribution among the three studied implants (PFNA, InterTAN, and MIN). This may bring a promising implant for patients with UFTFs.

## Ethics statement

The study was conducted in compliance with the Declaration of Helsinki and was approved by the Institutional Review Board of Xi'an Hong Hui Hospital (approval number: 202305013).

## Funding

The research was funded via the Bureau project of Xi'an Health Commission (2024ms08), and the Clinical Application-oriented Medical Innovation Foundation (2021-NCRC-CXJJ-PY-23). The funding team never participated in research planning, conducting, collecting as well as data analysis.

## Consent to participate/Consent to publish

The volunteer has signed the informed consent before surgery and provided the consent to publish and report individual clinical data.

## Data availability statement

Data have been contained into this manuscript. Any further inquiry will be explained by corresponding authors.

## CRediT authorship contribution statement

**ChaoFeng Wang:** Writing – original draft, Investigation, Formal analysis. **MingMing Hou:** Validation, Supervision, Methodology. **CongMing Zhang:** Validation, Resources, Data curation. **Teng Ma:** Investigation, Funding acquisition, Formal analysis. **Zhong Li:** Project administration, Methodology. **Hua Lin:** Formal analysis, Data curation. **Kun Zhang:** Writing – review & editing, Software, Resources. **Qiang Huang:** Writing – review & editing, Data curation, Conceptualization.

## Declaration of competing interest

The authors declare that they have no known competing financial interests or personal relationships that could have appeared to influence the work reported in this paper.
